# Distribution Pattern of N6-Methyladenine DNA Modification in the Seashore Paspalum (*Paspalum vaginatum*) Genome

**DOI:** 10.3389/fpls.2022.922152

**Published:** 2022-07-07

**Authors:** Jiang-Shan Hao, Jian-Feng Xing, Xu Hu, Zhi-Yong Wang, Min-Qiang Tang, Li Liao

**Affiliations:** ^1^College of Tropical Crops, Hainan University, Haikou, China; ^2^Jinhua Polytechnic, Jinhua, China; ^3^Key Laboratory of Genetics and Germplasm Innovation of Tropical Special Forest Trees and Ornamental Plants, Ministry of Education, College of Forestry, Hainan University, Haikou, China

**Keywords:** Seashore paspalum, DNA 6mA modification, gene expression, stress resistance, transcription start site

## Abstract

N6-methyladenine (6mA) DNA modification has been detected in several eukaryotic organisms, in some of them, it plays important role in the regulation process of stress-resistance response. However, the genome-wide distribution patterns and potential functions of 6mA DNA modification in halophyte Seashore paspalum (*Paspalum vaginatum*) remain largely unknown. Here, we examined the 6mA landscape in the *P. vaginatum* genome by adopting single molecule real-time sequencing technology and found that 6mA modification sites were broadly distributed across the *P. vaginatum* genome. We demonstrated distinct 6mA methylation levels and 6mA distribution patterns in different types of transcription genes, which hinted at different epigenetic rules. Furthermore, the moderate 6mA density genes in *P. vaginatum* functionally correlated with stress resistance, which also maintained a higher transcriptional level. On the other hand, a specific 6mA distribution pattern in the gene body and near TSS was observed in gene groups with higher RNA expression, which maybe implied some kind of regularity between 6mA site distribution and the protein coding genes transcription was possible. Our study provides new insights into the association between 6mA methylation and gene expression, which may also contribute to key agronomic traits in *P. vaginatum.*

## Introduction

DNA modification is marketed as a kind of epigenetic inheritance (Heard and Martienssen, 2014; Allis and Jenuwein, 2016; Zhang H. et al., 2018; Wang et al., 2021), which contribute to the regulation of genome imprinting, transposon suppression, gene expression, embryonic development, and human tumor formation (Bergman and Cedar, 2013; Smith and Meissner, 2013; von Meyenn et al., 2016), without causing any change in DNA sequence itself (Heard and Martienssen, 2014; Allis and Jenuwein, 2016; Chachar et al., 2021). In the years, 6mA DNA methylation as an epigenetic marker has been gradually reported presenting in eukaryotes by multiple studies, involving in the special distribution patterns associated with potential functions of the important biological functions regulation, such as the regulation process of stress-resistance response (Lamke and Baurle, 2017; He and Li, 2018; Zhang Q. et al., 2018; Ma et al., 2019), embryonic development (Liang et al., 2018b) and tumorigenesis (Xiao et al., 2018).

Benefiting from the 6mA modification single-nucleotide-resolution-level detection performance of third-generation sequencing platform (Flusberg et al., 2010; Fang et al., 2012; Xiao et al., 2017), several studies have confirmed the genome-wide patterns of 6mA methylation, 6mA sites were relatively abundant located on gene bodies, and shared consensus sequence elements (motifs) in Arabidopsis, rice, soybean and strawberry (Liang et al., 2018b; Xie et al., 2020; Yuan et al., 2020). N6-methyladenine is also found to be enriched around TSS and then actively transcribed in gene expression in mammals and in some plants, such as Arabidopsis and rice (Liang et al., 2020). These signs suggest that 6mA may exert a regulatory impact on gene expression in eukaryotes related to biological processes by its remarkable genome-wide patterns. It seems clear that monitoring such modifications will continue to broaden our knowledge about gene regulation and epigenetic inheritance in plants.

Seashore paspalum (*Paspalum vaginatum* Swartz) (2n= 2×= 20) has been utilized as turf for almost one hundred years (Wu et al., 2018; Qi et al., 2019), which is an important halophytic warm-seasoned perennial grass widely used on athletic fields, golf course, and landscape areas in tropical and subtropical areas (Liu et al., 2017; Wu et al., 2020). Because of its strong salt tolerance, *P. vaginatum* can even be watered with sea water and especially important in locations near the sea or the regions with water quality issues. Related studies have shown that *P. vaginatum* can maintain photosynthesis, stolon growth rate, and tissue water content under salt stress conditions through osmotic adjustment (Lee et al., 2004; Liu et al., 2011). In view of 6mA is positively correlated with the expression of key stress-related genes and contributes to key agronomic traits in *Oryza sativa* (Zhang Q. et al., 2018), we were interested in the question of whether in *P. vaginatum* there was a connection between the genome-wide distribution patterns of 6mA DNA modification and its salt tolerance regulation.

Thus, in this study, we detected 6mA DNA methylation sites based on PacBio SMRT sequencing technology, aiming to reveal high-quality 6mA methylomes in *P. vaginatum*; furthermore, we described the distribution detail of 6mA modification in different types of genes and showed that various 6mA distribution states were observed in these types of genes. Besides, we demonstrated specific 6mA distribution pattern was associated with actively expressed genes and protein coding genes with medium 6mA methylation level might relate to salt tolerance of *P. vaginatum*.

## Materials and Methods

### Identification of 6mA in the *Paspalum vaginatum* Genome

The genomic SMRT sequencing reads raw data of *P. vaginatum* were acquired by our research group in the early stages but haven’t been officially published. N6-methyladenine DNA modifications of *P. vaginatum* genome were detected by the PacBio SMRT analysis platform (version 2.3.0)^1^ (Xiao et al., 2017). The detailed analysis workflows were as follows: the raw reads were initially aligned to the corresponding reference genome using pbalign with the parameters -seed = 1-minAccuracy = 0.75 – minLength = 50 –concordant – algorithmOptions = “- useQuality” – algorithmOptions = “- minMatch 12 -bestn 10 - minPctIdentity 70.0.” The polymerase kinetics information was loaded following alignment by loadChemistry.py and loadPulses scripts with parameters “-metrics DeletionQV, IPD, InsertionQV, PulseWidth, QualityValue, MergeQV, SubstitutionQV, DeletionTag.” Then cmph5tools was used to sort the post-aligned datasets. Finally, 6mA modification was identified by using ipdSummary.py script with parameters “- methylFraction - identify 6mA - numWorkers 4”; 6mA sites with coverage of no less than 25× were screened out for further analysis.

### Whole Genomic Distribution and Conservative Motif of 6mA

According to the methylation levels at each 6mA site across the genome, we divided the 6mA sites into three fractions: low (0–35%), medium (35–65%), and high (65–100%). Circos (Krzywinski et al., 2009) was used to depict 6mA site density of the three types across all chromosomes by every 20 Kbp length. The density of 6mA was calculated by the ratio of the number of 6mA sites to the number of A bases in the region.

The genome of *P. vaginatum* was divided into the protein-coding gene, non-coding RNA gene, and intergenic regions; the protein-coding gene region was further subdivided into 5’UTR, CDS, 3’UTR, and intron. The number and density of the 6mA methylation sites in these regions and the expected 6mA were counted and calculated, respectively. The binomial test was used to observe the significance of the difference between the 6mA density and the expected 6mA density in each gene characteristic region. The 6mA density was calculated by the ratio of the number of 6mA sites in the region to the number of A bases in the region.

Then, we extracted 20 bp on the upstream and downstream flanks of each 6mA modification to predict the conserved sequence motifs, by using STREME 5.3.3 (Bailey et al., 2009) with the parameters “-minw 6 -maxw 15 -kmer 3.” The corresponding *p* values of the motifs were calculated using *Fisher’s* exact test.

### Various Distribution Characters of 6mA in Genes

We counted the content of methylated genes and the different 6mA methylation site frequency in eight types of genes, such as protein-coding genes, and four non-coding genes (miRNA, rRNA, snRNA, tRNA), and three types of transposon genes (LINE, LTR, SINE). The 6mA density of these genes was subjected to multiple comparisons by the LSD method, and Bonferroni correction was used to correct the *p* value, and the significance level of 0.01 was perceived as statistically different.

To evaluate and compare 6mA distribution patterns between various types of genes, we normalized the length of each gene and described the 6mA site distribution of eight types of genes in every 10% length. Furthermore, we plotted the 6mA number and 6mA occupancy in the 5K bp region of the upstream and downstream TSS by 50 bp as unit. The 6mA occupancy rate indicates the percentage of the number of genes containing 6mA in the unit to the total number of genes.

### Gene Function Analysis of Different 6mA Density Level Genes

All protein-coding genes were classified into four groups according to the parameter *K* = log_2_(FC), FC is the fold change between the 6mA density of the gene and the average value: high 6mA density (*K* > 1), moderate 6mA density (−1 > *K* > 1), low 6mA density (*K* < −1), and non-6mA-modified. These four groups of genes performed enrichment analysis based on GO annotation information and drew the GO functional classification map using WEGO (Ye et al., 2018), Chi-square tests were carried out for all datasets of particular GO terms. *P*-values were obtained to indicate the sample differences, and the sample difference was considered significant when the *p* < 0.05.

### Computational Analysis of 6mA Density and Gene Expression

RNAs from the leaf of *P. vaginatum* were extracted by the RNeasy Plus Mini Kit (Qiagen). We generated the cDNA libraries using the NEBNext Ultra RNA Library Prep Kit (Neb). The quantified libraries were then prepared for sequencing on the Illumina HiSeq X-ten sequencing platform. After removing the adapter and primer sequences, low-quality reads with more than 20% low-quality bases (quality < 20) were filtered out. The abundance of gene expression was calculated by cufflinks version 2.2.1 (Trapnell et al., 2010) using the fragments kilobase of exon model per million mapped reads (FPKM). All the genes with FPKM value and 6mA density larger than zero were selected for analysis. We used R version 3.6.1 to perform the statistical analysis and prepare figures, all significant differences were assessed by Student’s *t*-test.

## Results

### Overview of 6mA Modifications in the *Paspalum vaginatum* Genome

**FIGURE 1:**
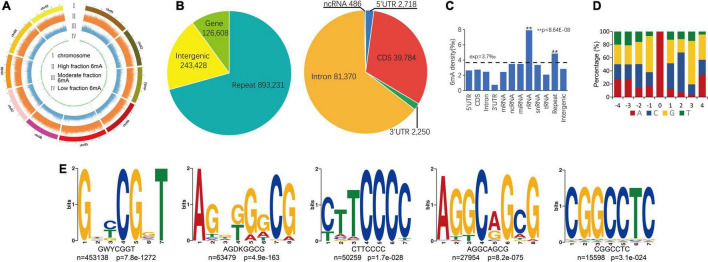
Overview of 6mA in global genome. **(A)** 6mA distribution mapping across chromosomes. **(B)** 6mA sites in different genomic regions. **(C)** 6mA densities in different regions. **(D)** Content percentage of the bases A, T, C, and G in the upstream and downstream 4 bp of 6mA sites (position “0”). **(E)** The motif sequences of 6mA detected by STREME assay, the name of the motif, the number of positive sequences matching the motif (n) and the corresponding *p*-value generated by STREME are shown under the logo. ^**^ means statistical extreme significant difference.

About 969,960 6mA modification sites were detected widely in *P. vaginatum* genome based on PacBio SMRT sequencing data, distributed evenly across all 10 chromosomes (Figure 1A and Supplementary Information 1). The 6mA density of each chromosome ranged from 0.34 to 0.41%. The difference in 6mA distribution between positive and negative strands was smaller than the difference between chromosomes (Table 1). The density of the 6mA (6mA/A) across the whole genome was approximately 0.37%, which was similar to the *Chlamydomonas reinhardtii* (∼ 0.4%) (Fu et al., 2015), but higher than 6mA density of *Arabidopsis thaliana* (0.0048%) (Liang et al., 2018b), *Fragaria vesca* (0.139%) (Xie et al., 2020) and soybeans (0.0399%, 0.0406%) (Yuan et al., 2020), and lower than that previously detected in *C. elegans* (∼0.7%) (Greer et al., 2015) and *Hesseltinella vesiculosa* (2.8%) (Mondo et al., 2017). Furthermore, we divided the 969,960 6mA sites into three fractions: low (0–35%), medium (35–65%), and high (65–100%), which were plotted in Figure 1A, and we found low-frequency methylation sites were significantly less than high frequency and medium frequency sites, and high-frequency methylation sites were higher than the other two types of methylation site frequencies. We investigated the number of 6mA sites in different regions of the *P. vaginatum* genome, the result demonstrated that most of the 6mA modification sites were located in the repeated sequence (Figure 1B and Supplementary Table 1). After comparing the density of 6mA in each genome region with the expected value, we found that 6mA was enriched in the rRNA and repeated sequence (Figure 1C and Supplementary Table 1).

**TABLE 1:** Density of N6-methyladenine (6mA) across the *Paspalum vaginatum* genomic DNA.

	Size	No. A bases (−)	No. A bases (+)	No. A bases	No. 6mA sites (−)	No. 6mA sites (+)	No. 6mA sites	Density (−)	Density (+)	Density
chr01	58,590,413	15,780,524	15,765,748	31,546,272	54,240	53,919	108,159	0.34%	0.34%	0.34%
chr02	48,219,515	13,108,330	13,059,912	26,168,242	45,033	45,048	90,081	0.34%	0.34%	0.34%
chr03	46,211,395	12,417,356	12,415,619	24,832,975	42,662	43,454	86,116	0.34%	0.35%	0.35%
chr04	48,327,047	13,053,919	13,040,765	26,094,684	48,978	50,718	99,696	0.38%	0.39%	0.38%
chr05	61,355,573	16,825,528	16,828,116	33,653,644	59,183	59,502	118,685	0.35%	0.35%	0.35%
chr06	45,010,524	12,085,996	12,135,716	24,221,712	45,848	45,313	91,161	0.38%	0.37%	0.38%
chr07	43,553,415	11,824,538	11,839,346	23,663,884	44,629	44,482	89,111	0.38%	0.38%	0.38%
chr08	43,423,328	11,846,570	11,827,008	23,673,578	49,289	48,611	97,900	0.42%	0.41%	0.41%
chr09	47,058,851	12,782,768	12,721,317	25,504,085	49,616	50,243	99,859	0.39%	0.39%	0.39%
chr10	43,887,791	11,830,548	11,825,941	23,656,489	44,691	44,501	89,192	0.38%	0.38%	0.38%
total	485,637,852	131,556,077	131,459,488	263,015,565	484,169	485,791	969,960	0.37%	0.37%	0.37%

To determine whether consensus sequence content elements were shared around the identified 6mA sites, we surveyed the occurrence frequency of four bases in the upstream and downstream 4-bp sequences from the 6mA sites. Unlike the core sequence pattern (GAG) within the sequence flanking the 6mA sites in humans (Xiao et al., 2018) or *Fragaria vesca* (Xie et al., 2020), we only observed the high probability (55%) of guanine (G) in upstream 1 bp of the sites (Figure 1D). We also found that cytosine (C) and G exist with high frequency in downstream 2 and 3 bp of the sites (Figure 1D). We extended the flanking regions around the 6mA sites and extracted the upstream and downstream 20-bp sequences from the 6mA sites, to further performed an unbiased search for significant consensus motifs using STREME (Bailey, 2021). Five prominent motif sequences were detected and the GWYCGGT motif was significantly enriched (Figure 1E). The AGGCAGCG motif is include AGG which has been detected close to the 6mA sites among multiple species genomes (Greer et al., 2015; Xiao et al., 2018; Xie et al., 2020).

### Non-sparse Distribution of 6mA Sites and Most Methylated Gene Rate in Protein Coding Genes

**FIGURE 2:**
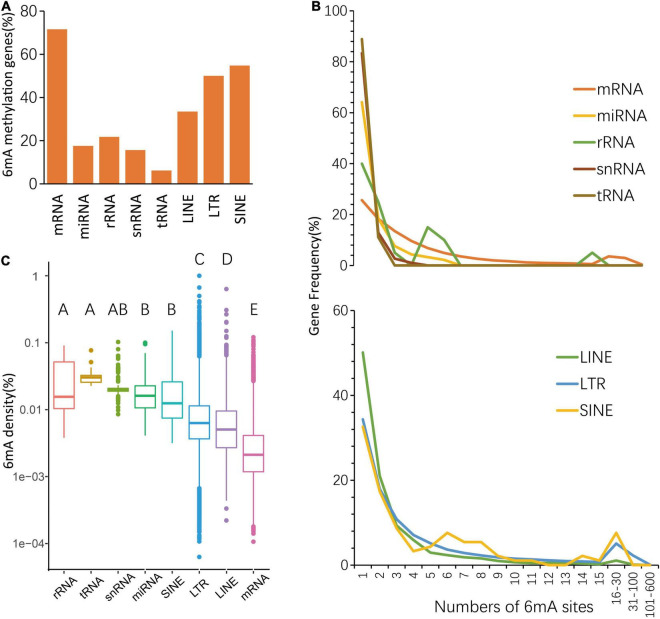
Distribution of 6mA in echo type of genes. **(A)** The percentage of 6mA-methylated genes. **(B)** Statistics of gene numbers with different 6mA sites. **(C)** The different 6mA modification density in echo type of genes.

To further understand different 6mA distribution patterns among multiple types of genes, including protein-coding RNA (mRNA), non-coding RNA, and three types of transposon genes, such as LINE (Long interspersed nuclear elements), LTR (long terminal repeat), SINE (short interspersed nuclear elements), firstly we counted the number of genes with 6mA sites, and result shows 19,734 mRNA genes (71.55%) are harboring 6mA modification sites, of which methylation rate was the highest (Figure 2A and Supplementary Table 2). Furthermore, the 6mA site numbers within each gene were surveyed and we found that the enrichment of 6mA sites in all types of genes revealed a similar trend, that half of 6mA methylated genes were only with one to three sites (Figure 2B and Supplementary Table 3). Interestingly, the curve representing the quantitative distribution of mRNA genes with a specific 6mA site number was gentlest among multiple types of genes (Figure 2B and Supplementary Table 3), which suggested the genes with multiple sites have greater weight in mRNA genes than other types of genes. However, non-coding genes seem to have higher methylation density, while protein coding genes have the lowest methylation density (Figure 2C and Supplementary Information 2). The above results indicate that higher number of 6mA sites but a lower 6mA density in mRNA which might be caused by the higher adenine content (Supplementary Table 1).

### Specific 6mA Distribution Pattern in Protein Coding Genes

**FIGURE 3:**
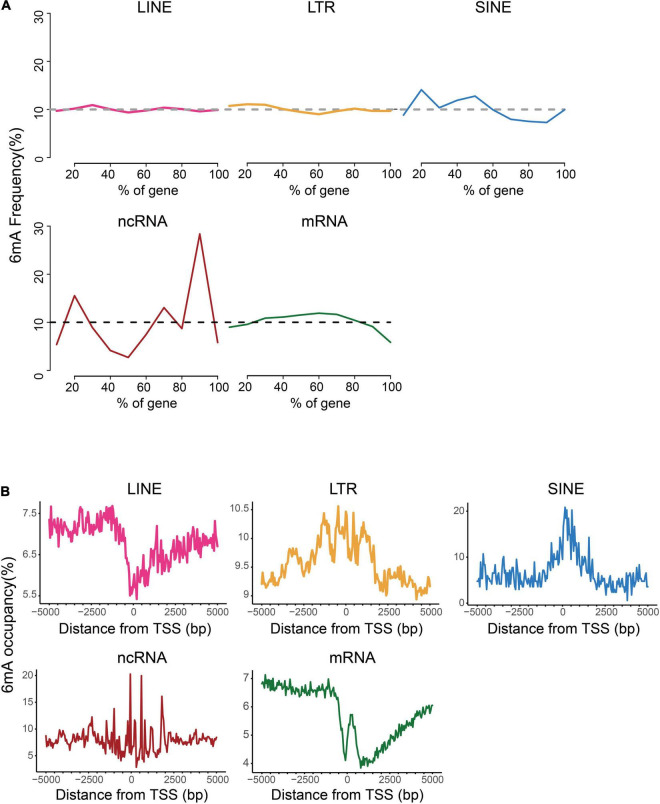
Distribution patterns of 6mA in echo type of genes. **(A)** Frequency of 6mA sites at relative positions in gene bodies. **(B)** Distribution of 6mA occupancy around the transcription start site (TSS).

For further investigation on the distinction of 6mA distribution among these types of genes, we analyzed the distribution of 6mA sites in the gene body and found that 6mA were evenly distributed across the gene body in LINE and LTR, but enriched at both ends of non-coding RNA gene body. More strikingly, the 6mA site in the coding gene was more prone in the middle of the gene but reductive at both ends (Figure 3A). Previous studies showed that the 6mA enrichment in TSS (transcription start site) region could activate gene expression (Liang et al., 2018c; Xiao et al., 2018). Therefore, we plotted the distribution pattern of 6mA sites in the TSS of the five types of genes. The 6mA occupancy represented the relative number of genes containing 6mA sites against the total number of corresponding type genes. We determined the number of 6mA-methylated genes in consecutive 50-bp windows throughout the 5-kb regions upstream and downstream of the TSS (Figure 3B). On LTR, SINE, and ncRNA genes, the closer to the TSS site, the more prone to 6mA methylation enrichment, particularly of the SINE genes and ncRNA genes, approximately 20% were methylated near TSS site. By contrast, on the LINE and coding RNA, there was a significant reduction of 6mA modification near the TSS site, and a sharp increase of 6mA enrichment peak within 500 bp downstream of the TSS region of coding RNA, especially this trend was more obvious in mRNA.

### Genes With Moderate Methylation Levels Enriched the Functions Related to Stress Resistance

**FIGURE 4:**
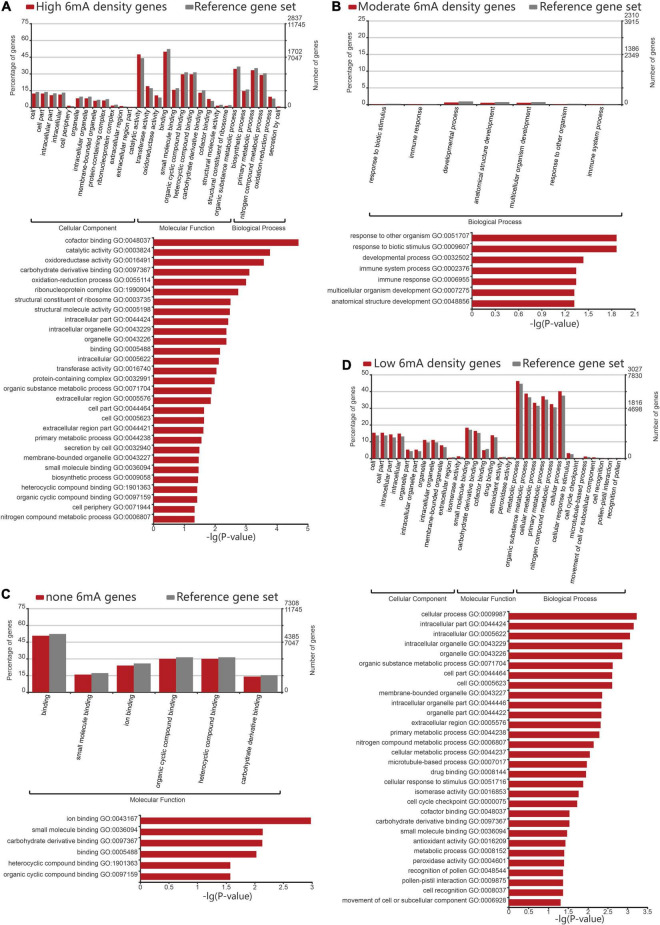
Gene ontology analysis of different 6mA methylation levels genes. **(A)** Gene ontology analysis of high density 6mA genes. **(B)** Gene ontology analysis of medium density 6mA genes. **(C)** Gene ontology analysis of non-6mA-modified genes. **(D)** Gene ontology analysis of low density 6mA genes.

In order to assess the 6mA preference for gene function in *P. vaginatum*, we performed GO enrichment analysis for protein-coding genes classified into four grades by 6mA density. The result revealed that the non-6mA-modified genes were mainly enriched in molecular function, mainly related to ion binding, small molecule binding, carbohydrate derivative binding, and compound binding (Figure 4C and Supplementary Table 7). On the other hand, genes with high 6mA density and low 6mA density were both enriched in the catalogs involving cell components, molecular functions, and biological processes (Figures 4A,D and Supplementary Tables 4, 6). In detail, the functions of high 6mA density genes enriched in cell components were mainly related to the formation of organelles, and molecular functions were related to catalytic activity, redox activity, transferase activity, and binding of the cyclic compound, coenzyme factors, and carbohydrate derivative, while the biological process was mainly enriched in organic matter metabolism, biosynthesis, primary metabolism, nitrogen compound metabolism and other functions (Figure 4A and Supplementary Table 4). Low 6mA density genes shared a similar gene function with high-density genes in cellular components, which was associated with intracellular substances or cell internal organs; in terms of molecular function, low 6mA density genes were related to the binding of small molecular substances, carbohydrate derivatives, coenzyme factors; while enriched in biological processes, they were related to organic metabolism, cell metabolism, primary metabolism, and nitrogen compound metabolism (Figure 4D and Supplementary Table 6). The moderate 6mA density genes were mainly enriched in biological processes, which are mainly related to stress resistance such as response to a biotic stimulus or other organisms, immune response, immune system process, as well as anatomical structure development (Figure 4B and Supplementary Table 5). Given the published viewpoint that the special physiological structure of *P. vaginatum*, adaxial leaf papillae, contributed to salt tolerance (Spiekerman and Devos, 2020), the gene function of anatomical structure development might serve to develop the salt-resistant characteristic of *P. vaginatum* together with stress resistant function showed in moderate 6mA density genes by GO analysis. It implied genes related to stress resistance including salt tolerance in *P. vaginatum* were perhaps controlled by particular epigenetic regulation mechanism which needs to maintain a moderate 6mA methylation level.

### Higher Transcriptional Level in Moderate Methylation Level Genes and a More Obvious 6mA Distribution Pattern in Higher Transcriptional Level Genes

**FIGURE 5:**
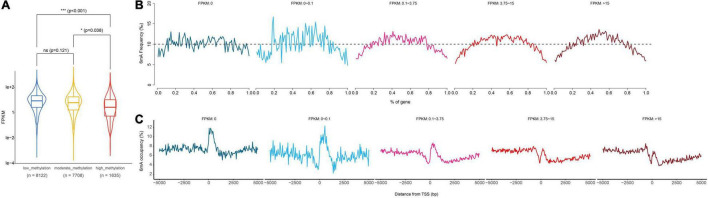
Relationship between gene transcription and 6mA methylation. **(A)** Computational analysis of gene expression in different methylation levels genes. **(B,C)** Distribution of 6mA occupancy around the transcription start site in different methylation levels genes. * means statistical significant difference, *** means statistical extreme significant difference and *p*-value < 0.001.

Previous studies have shown that 6mA was strongly associated with gene activation or suppression in animals, plants, and fungi, and suggested that 6mA was an epigenetic marker regulating gene expression in eukaryotes. Therefore, in order to explore the correlation between 6mA distribution and gene expression in paspalum, we compared the gene expression of high 6mA density group, medium 6mA density group, and low 6mA density group, and found that the transcription level of high 6mA density group was significantly lower than that of medium and low 6mA density group (Figure 5A and Supplementary Information 2). In addition, genes with different expression levels were grouped and the distribution characteristics of 6mA in the gene body and near TSS were statistically analyzed (Figures 5B,C). We found that although less distributed at the 5′ and 3′ ends of all types of genes, 6mA showed a tendency to be enriched in the middle of the expressed genes, and this characteristic was more obvious in the high expressed genes, compared with the uniform distribution of 6mA in the middle of the low or no expressed genes (FPKM < 0.01) (Figure 5B). The difference in 6mA distribution between high expression and low expression or no expression genes was also reflected in the distribution of 6mA near TSS. All the expressed genes showed a trend of sharp decline and sharp rise near TSS, especially in the highly expressed genes, which not only retained this distribution trend but also gradually formed a binomial peak distribution.

## Discussion

The recent extensive research on 6mA DNA modification in several eukaryotic genomes opens a new and promising territory for epigenetic research (Liang et al., 2018a). The epigenetic roles of 6mA in eukaryotes play regulatory functions and are essential for eukaryotic development by being associated with gene expression, stress responses, and epigenetic memory maintenance in plants (Greer et al., 2015; Liang et al., 2018a; Zhang Q. et al., 2018). Genome-wide 6mA distributions in land plants have been reported in *A. thaliana*, rice, woodland strawberry, and soybeans (Liang et al., 2018b; Zhang Q. et al., 2018; Xie et al., 2020; Yuan et al., 2020). In this study, we identified the genome-wide 6mA sites at single-nucleotide resolution in *P. vaginatum* genomes using the SMRT sequencing and assembled high-quality reference genomes. The result showed 969,960 6mA sites broadly distributed across the *P. vaginatum* genome, most of which were medium and high-frequency methylation sites and focused on the repeating fragment area.

After investigating the distribution characteristic of 6mA modification in *P. vaginatum*, we found a significant enrichment arose around the transcription start sites (TSSs). In particular, the coding gene showed that 6mA are highly enriched around TSS with a bimodal distribution obvious. It was very similar to the binomial distribution pattern of 6mA in the genomic of *Chlamydomonas reinhardtii*, in which researchers demonstrated that the region-specific bimodal and strongly periodic distribution pattern of 6mA around TSS contributed to nucleosome positioning and transcription initiation (Fu et al., 2015). Furthermore, a middle-prone pattern was occurred in gene body, likewise, more obvious in genes with high expression. This distribution pattern was also shown in rice and *F. vesca* (Zhang Q. et al., 2018; Xie et al., 2020). Collectively, these findings indicated a positively association between this distribution pattern and gene expression. We speculated that there may be two possibilities for explaining this association: first, the mechanism of transcription initiation that occurred around TSS was maintained for following exon transcript by the enrichment of 6mA sites in the middle of the gene body; second, 6mA might be probably involved in the accurate exon recognition just like 5mC in Chinese pine (Niu et al., 2022). Although there are sufficient evidence to prove the association between 6mA of the gene body and gene expression, the regulation mechanism still deserves more experiments and data to further investigation.

On the other hand, although the methylation density of protein coding genes had lower value, which was different from other organisms (Liang et al., 2018b; Xiao et al., 2018; Zhang Q. et al., 2018; Xie et al., 2020), some of them still maintain more than three 6mA sites (Figure 2B) due to high adenine content (Supplementary Table 1), which means single individual gene maybe provide more chance to accept the regulations from 6mA than other type genes. In general, its highest methylated gene rate and the special 6mA distribution pattern near TSS and in the gene body all suggested the possibility that 6mA epigenetic regulation may be a widely adopted mechanism for protein coding genes in *P. vaginatum*. r

It’s interesting that most 6mA sites were located in the repeat sequence region (Figure 1) which also contained most adenine (Supplementary Table 1), and the methylation density of the repeat sequence region was higher than that of the protein coding genes region, but genes with sparse 6mA sites were still more enrich in repeat sequence region than that in protein coding genes region (Figure 2B). Considering we counted gene methylation density without non-6mA-modified genes, these findings may hint at a preference occurrence mechanism of 6mA in repeat sequence region that 6mA sites tended to distribute on repeat sequence genes with low content of adenine, which resulted in enrichment of genes with less 6mA sits but maintained higher 6mA density of global repeat sequence region than protein coding genes. The above results showed that repeat sequence genes were perhaps regulate by different epigenetic rule than protein coding genes.

A significant percentage of studies about 6mA showed the association between this type of DNA methylation and gene expression, which was consistent with the findings in this study. At that point, we reflected on if genes with 6mA have preferences in the biological function. The GO analysis for protein coding gene groups with four grades of 6mA density revealed that different biological functions might be regulated by different methylation levels. Given salt sensitivity of halophytic *P. vaginatum* seemed to be attributed to its ability that maintain greater levels of photosynthesis, osmotic adjustment, shoot growth rates, and tissue water content (Lee et al., 2004; Liu et al., 2012; Katuwal et al., 2020), as well as special physiological structure of leaf (Spiekerman and Devos, 2020), its excellent stress resistance perhaps requires the coordinated function of mass essential genes involving all four grades of 6mA density, but the gene function of the moderate 6mA density genes group showed by GO analysis still drew the most attention for the possibility that these genes maybe contribute to its stress resistance dominantly, which deserves further investigation. Interestingly, 6mA levels of genes in rice were demonstrated to be associated with temperature stress (Zhang Q. et al., 2018), and in *F. vesca*, we also observed the preferences on the biological function among genes with different 6mA density levels, which may be associated with the response of environmental stress (Xie et al., 2020). Based on these discoveries, we hypothesized that 6mA may be may serve as an epigenetic marker that mediates capacity of stress responses by different distribution density pattern in these organisms.

Based on transcriptomics data, we found protein coding gene groups with medium 6mA density or low 6mA density had higher RNA expression values (Figure 5A). Although inconsistent with conclusions found in a series of reports (Fu et al., 2015; Greer et al., 2015; Mondo et al., 2017; Xiao et al., 2018; Zhang Q. et al., 2018; Xie et al., 2020), this result was agreed with research in mammalian embryonic stem cells (Wu et al., 2016), mouse brain (Yao et al., 2017) and Mammalian Mitochondrial DNA (Hao et al., 2020). In view of the association between gene function of the moderate 6mA density genes group and the plant character of stress resistance, moderate 6mA density genes with higher expression levels might reflect the requirement for salt tolerance of *P. vaginatum*. What is more interesting is that in genes groups with higher RNA expression we observed a specific 6mA distribution pattern in the gene body and near TSS (Figures 5B,C), which was in accordance with a similar distribution pattern reported in previous research (Fu et al., 2015; Liang et al., 2018b; Xiao et al., 2018; Zhang Q. et al., 2018; Xie et al., 2020). The distribution characteristic of 6mA observed in highly expressed genes perhaps reflected some kind of regularity between 6mA methylation and the protein coding genes transcription. In summary, the results we demonstrated in this study revealed the connection between the 6mA methylation level and resistance of *P. vaginatum*, as well as the 6mA distribution pattern and the protein coding genes transcription, which hint at a possibility of the regulatory mechanism of plant resistance by 6mA methylation.

## Data Availability Statement

The original contributions presented in this study are included in the article/Supplementary Material, further inquiries can be directed to the corresponding authors.

## Author Contributions

LL and M-QT contributed to the conception and design of the study. J-SH and J-FX detected 6mA. J-SH, J-FX, and XH performed the statistical analysis. J-SH wrote the first draft of the manuscript. LL, M-QT, J-SH, J-FX, XH, and Z-YW wrote sections of the manuscript. All authors contributed to the manuscript revision, read, and approved the submitted version.

## Conflict of Interest

The authors declare that the research was conducted in the absence of any commercial or financial relationships that could be construed as a potential conflict of interest.

## Publisher’s Note

All claims expressed in this article are solely those of the authors and do not necessarily represent those of their affiliated organizations, or those of the publisher, the editors and the reviewers. Any product that may be evaluated in this article, or claim that may be made by its manufacturer, is not guaranteed or endorsed by the publisher.

## Footnotes

1
http://www.pacb.com/products-and/services/analytical-software/smrt-analysis/analysis-applications/epigenetics
