# Supplementary material for: Distribution Pattern of N6-Methyladenine DNA Modification in the Seashore Paspalum (Paspalum vaginatum) Genome
Source: Front Plant Sci. 2022 Jul 7;13:922152. doi: 10.3389/fpls.2022.922152 (PMC9302377; doi:10.3389/fpls.2022.922152)
Supplement: Supplementary file 1 [file Data_Sheet_1.zip › Supplementary material tables.docx]

Table S1. 6mA distribution in genomic features

| Genome feature | No. A bases | No. 6mA sites | Density(‰) | binomial test-p |
| --- | --- | --- | --- | --- |
| 5'UTR | 1,030,737 | 2,718 | 2.64 | 2.20E-16 |
| CDS | 14,525,359 | 3,9784 | 2.74 | 2.20E-16 |
| Exon | 18,584,680 | 4,4752 | 2.41 | 2.20E-16 |
| Intron | 33,108,392 | 81,370 | 2.46 | 2.20E-16 |
| 3'UTR | 3,028,584 | 2,250 | 0.74 | 2.20E-16 |
| mRNA | 51,693,072 | 126,122 | 2.44 | 2.20E-16 |
| ncRNA | 139,762 | 486 | 3.48 | 0.179 |
| miRNA | 45,102 | 157 | 3.48 | 4.85E-01 |
| rRNA | 7,977 | 63 | 7.90 | 8.64E-08 |
| snRNA | 67,453 | 226 | 3.35 | 1.45E-01 |
| tRNA | 19,230 | 40 | 2.08 | 8.34E-05 |
| Repeat | 184,305,288 | 893,231 | 4.85 | 2.20E-16 |
| Intergenic | 85851762 | 243428 | 2.84 | 2.20E-16 |

Table S2. 6mA methylation gene ratio of echo type of genes

| Gene type | No. Total genes | No. 6mA genes | Frequency (%) |
| --- | --- | --- | --- |
| mRNA | 19734 | 27580 | 71.55 |
| miRNA | 92 | 523 | 17.59 |
| rRNA | 20 | 92 | 21.74 |
| snRNA | 186 | 1189 | 15.64 |
| tRNA | 36 | 580 | 6.21 |
| LINE | 4681 | 13980 | 33.48 |
| LTR | 139586 | 278952 | 50.04 |
| SINE | 92 | 168 | 54.76 |

Table S3. The 6mA site distribution of echo type of genes

| No. 6mA sites/gene | LINE | LTR | SINE | miRNA | mRNA | rRNA | snRNA |
| --- | --- | --- | --- | --- | --- | --- | --- |
| 1 | 2345 | 47932 | 30 | 59 | 5062 | 8 | 155 |
| 2 | 985 | 25090 | 16 | 17 | 3611 | 5 | 24 |
| 3 | 437 | 15131 | 8 | 7 | 2666 | 1 | 5 |
| 4 | 277 | 9918 | 3 | 4 | 1904 | 0 | 2 |
| 5 | 138 | 7173 | 4 | 3 | 1349 | 3 | 0 |
| 6 | 110 | 5091 | 7 | 2 | 973 | 2 | 0 |
| 7 | 86 | 3971 | 5 | 0 | 703 | 0 | 0 |
| 8 | 74 | 3188 | 5 | 0 | 517 | 0 | 0 |
| 9 | 46 | 2560 | 2 | 0 | 391 | 0 | 0 |
| 10 | 31 | 2144 | 1 | 0 | 322 | 0 | 0 |
| 11 | 23 | 1863 | 1 | 0 | 234 | 0 | 0 |
| 12 | 24 | 1569 | 0 | 0 | 203 | 0 | 0 |
| 13 | 22 | 1352 | 0 | 0 | 178 | 0 | 0 |
| 14 | 17 | 1204 | 2 | 0 | 145 | 0 | 0 |
| 15 | 10 | 1001 | 1 | 0 | 106 | 1 | 0 |
| 16-30 | 50 | 7101 | 7 | 0 | 704 | 0 | 0 |
| 31-100 | 6 | 3153 | 0 | 0 | 580 | 0 | 0 |
| 101-600 | 0 | 143 | 0 | 0 | 86 | 0 | 0 |

Table S4. gene ontology enrichment category of genes with high 6mA level

| GO_ID | Ontology | Gene number  (Target /background) | Gene percentage (  Target /background) | P_Value | Term |
| --- | --- | --- | --- | --- | --- |
| GO:0005623 | Cellular Component | 465:2145 | 12.3:13.7 | 0.023 | cell |
| GO:0044464 |  | 465:2145 | 12.3:13.7 | 0.023 | cell part |
| GO:0044464 |  | 465:2145 | 12.3:13.7 | 0.023 | cell part |
| GO:0044424 |  | 409:1962 | 10.8:12.5 | 0.004 | intracellular part |
| GO:0005622 |  | 433:2045 | 11.4:13.1 | 0.008 | intracellular |
| GO:0071944 |  | 52:157 | 1.4:1.0 | 0.046 | cell periphery |
| GO:0043226 |  | 304:1493 | 8.0:9.5 | 0.004 | organelle |
| GO:0043229 |  | 304:1493 | 8.0:9.5 | 0.004 | intracellular organelle |
| GO:0043227 |  | 218:1051 | 5.8:6.7 | 0.034 | membrane-bounded organelle |
| GO:0032991 |  | 232:1147 | 6.1:7.3 | 0.010 | protein-containing complex |
| GO:1990904 |  | 56:360 | 1.5:2.3 | 0.002 | ribonucleoprotein complex |
| GO:0005576 |  | 42:112 | 1.1:0.7 | 0.014 | extracellular region |
| GO:0044421 |  | 11:20 | 0.3:0.1 | 0.024 | extracellular region part |
| GO:0044421 |  | 11:20 | 0.3:0.1 | 0.024 | extracellular region part |
| GO:0003824 | Molecular Function | 1793:6892 | 47.4:44.0 | <0.001 | catalytic activity |
| GO:0016740 |  | 715:2679 | 18.9:17.1 | 0.009 | transferase activity |
| GO:0016491 |  | 401:1363 | 10.6:8.7 | <0.001 | oxidoreductase activity |
| GO:0005488 |  | 1884:8182 | 49.8:52.2 | 0.007 | binding |
| GO:0036094 |  | 592:2675 | 15.6:17.1 | 0.035 | small molecule binding |
| GO:0097159 |  | 1117:4896 | 29.5:31.3 | 0.038 | organic cyclic compound binding |
| GO:1901363 |  | 1117:4896 | 29.5:31.3 | 0.038 | heterocyclic compound binding |
| GO:0097367 |  | 493:2379 | 13.0:15.2 | 0.001 | carbohydrate derivative binding |
| GO:0048037 |  | 281:877 | 7.4:5.6 | <0.001 | cofactor binding |
| GO:0005198 |  | 54:341 | 1.4:2.2 | 0.003 | structural molecule activity |
| GO:0003735 |  | 44:291 | 1.2:1.9 | 0.003 | structural constituent of ribosome |
| GO:0071704 | Biological Process | 1300:5720 | 34.4:36.5 | 0.013 | organic substance metabolic process |
| GO:0009058 |  | 549:2489 | 14.5:15.9 | 0.036 | biosynthetic process |
| GO:0044238 |  | 1256:5497 | 33.2:35.1 | 0.028 | primary metabolic process |
| GO:0006807 |  | 1092:4780 | 28.9:30.5 | 0.047 | nitrogen compound metabolic process |
| GO:0055114 |  | 356:1219 | 9.4:7.8 | 0.001 | oxidation-reduction process |
| GO:0032940 |  | 26:66 | 0.7:0.4 | 0.032 | secretion by cell |

Table S5. gene ontology enrichment category of genes with medium 6mA level

| GO_ID | Ontology | Gene number  (Target /background) | Gene percentage (  Target /background) | P_Value | Term |
| --- | --- | --- | --- | --- | --- |
| GO:0009607 | Biological Process | 9:37 | 0.1:0.2 | 0.014 | response to biotic stimulus |
| GO:0006955 |  | 5:22 | 0.1:0.1 | 0.045 | immune response |
| GO:0032502 |  | 59:138 | 0.6:0.9 | 0.037 | developmental process |
| GO:0048856 |  | 48:114 | 0.5:0.7 | 0.048 | anatomical structure development |
| GO:0007275 |  | 44:106 | 0.5:0.7 | 0.048 | multicellular organism development |
| GO:0051707 |  | 9:37 | 0.1:0.2 | 0.014 | response to other organism |
| GO:0002376 |  | 5:22 | 0.1:0.1 | 0.045 | immune system process |

Table S6. gene ontology enrichment category of genes with low 6mA level

| GO_ID | Ontology | Gene number  (Target /background) | Gene percentage (  Target /background) | P_Value | Term |
| --- | --- | --- | --- | --- | --- |
| GO:0005623 | Cellular Component | 926:2145 | 15.3:13.7 | 0.002 | cell |
| GO:0044464 |  | 926:2145 | 15.3:13.7 | 0.002 | cell part |
| GO:0044464 |  | 926:2145 | 15.3:13.7 | 0.002 | cell part |
| GO:0044424 |  | 863:1962 | 14.3:12.5 | 0.001 | intracellular part |
| GO:0005622 |  | 895:2045 | 14.8:13.1 | 0.001 | intracellular |
| GO:0044422 |  | 313:670 | 5.2:4.3 | 0.005 | organelle part |
| GO:0044446 |  | 313:670 | 5.2:4.3 | 0.005 | intracellular organelle part |
| GO:0043226 |  | 665:1493 | 11.0:9.5 | 0.001 | organelle |
| GO:0043229 |  | 665:1493 | 11.0:9.5 | 0.001 | intracellular organelle |
| GO:0043227 |  | 473:1051 | 7.8:6.7 | 0.004 | membrane-bounded organelle |
| GO:0044422 |  | 313:670 | 5.2:4.3 | 0.005 | organelle part |
| GO:0005576 |  | 23:112 | 0.4:0.7 | 0.005 | extracellular region |
| GO:0016853 | Molecular Function | 73:134 | 1.2:0.9 | 0.017 | isomerase activity |
| GO:0036094 |  | 1108:2675 | 18.3:17.1 | 0.034 | small molecule binding |
| GO:0097367 |  | 992:2379 | 16.4:15.2 | 0.030 | carbohydrate derivative binding |
| GO:0048037 |  | 294:877 | 4.9:5.6 | 0.029 | cofactor binding |
| GO:0008144 |  | 838:1966 | 13.8:12.6 | 0.011 | drug binding |
| GO:0016209 |  | 36:137 | 0.6:0.9 | 0.037 | antioxidant activity |
| GO:0004601 |  | 34:130 | 0.6:0.8 | 0.040 | peroxidase activity |
| GO:0008152 | Biological Process | 2795:6987 | 46.2:44.6 | 0.040 | metabolic process |
| GO:0071704 |  | 2346:5720 | 38.7:36.5 | 0.002 | organic substance metabolic process |
| GO:0044237 |  | 2011:4913 | 33.2:31.4 | 0.009 | cellular metabolic process |
| GO:0044238 |  | 2248:5497 | 37.1:35.1 | 0.005 | primary metabolic process |
| GO:0006807 |  | 1962:4780 | 32.4:30.5 | 0.007 | nitrogen compound metabolic process |

Table S7. gene ontology enrichment category of genes with non-6mA-level

| GO_ID | Ontology | Gene number  (Target /background) | Gene percentage (  Target /background) | P_Value | Term |
| --- | --- | --- | --- | --- | --- |
| GO:0005488 | Molecular Function | 4928:8182 | 50.6:52.2 | 0.009 | binding |
| GO:0036094 |  | 1539:2675 | 15.8:17.1 | 0.007 | small molecule binding |
| GO:0043167 |  | 2331:4033 | 23.9:25.8 | 0.001 | ion binding |
| GO:0097159 |  | 2918:4896 | 29.9:31.3 | 0.027 | organic cyclic compound binding |
| GO:1901363 |  | 2918:4896 | 29.9:31.3 | 0.027 | heterocyclic compound binding |
| GO:0097367 |  | 1361:2379 | 14.0:15.2 | 0.007 | carbohydrate derivative binding |
